# The effects of Juglone-Selenium combination on invasion and metastasis in pancreatic cancer cell lines

**DOI:** 10.4314/ahs.v22i2.37

**Published:** 2022-06

**Authors:** Hilal Arikoglu, Duygu Dursunoglu, Dudu Erkoc Kaya, Ebru Avci

**Affiliations:** 1 Selcuk University, Department of Medical Biology; 2 Selcuk University, Department of Histology and Embryology; 3 Necmettin Erbakan University, Medical Biology

**Keywords:** Pancreatic cancer cell lines, juglone-selenium, invasion, metastasis

## Abstract

**Background:**

Pancreatic cancer does not show any symptoms in the early period and metastatic process is already passed when the diagnosis is done. Therefore, in the battle with pancreatic cancer, novel treatment strategies, particularly antiinvasive and antimetastatic strategies, are needed. The cytotoxic and anticancer effects of juglone and sodium selenite (NaSe) have been showed in various cancer cells.

**Objectives:**

In this study, it is aimed to investigate the synergistic effects of juglone and selenium on PANC-1 and BxPC-3 pancreatic cancer cells.

**Methods:**

Antimetastatic effects of juglone-NaSe were carried out by adhesion and invasion assays and the genes and protein expressions. Expression analysis of the CDH1, ITGB3 and COL4A3 genes and their proteins E-cadherin, β3 integrin and tumstatin which play role in metastasis and angiogenesis processes, were done by qPCR and immunohistochemical analysis, respectively.

**Results:**

Study findings have provided evidences that the juglone-selenium has a cytotoxic and dose dependent suppressive effect on invasion and metastasis in PANC-1 and BxPC-3 cells.

**Conclusion:**

The juglone-NaSe has the potential to be a promising agent especially to inhibit invasion and metastasis in pancreatic cancer treatment. However, more in depth studies are needed to more clearly demonstrate the effects of juglone-selenium.

## Introduction

Cancer development is a multistage and versatile process. During this process, cancer cells continue to grow and spread to more areas by gaining new biochemical and molecular features. The role and importance of adhesion molecules, cytoskeleton, growth factors, angiogenesis factors, and molecules involved in the rearrangement of the matrix are the known facts in this metastatic process. In the treatment of cancer, early diagnosis is very important before metastatic process is beginning. Unfortunately, pancreatic cancer, as in some other cancers, does not show any symptoms in the early period and this process is already passed when the diagnosis is done. So, the average survival time in pancreatic cancer is shorter than 6 months, and the five year survival rate is reported as 9% 1. These rates are far below the survival rate of most common cancers. Although surgery continues to be a curative option for the early stages of the disease, only 15% of patients are suitable for surgical resection due to the localization of the pancreas, and the recurrence can be observed in 70–80% of resected tumors following surgery^2^. Treatment difficulties also arise in chemotherapy and/or radiotherapy due to the aggressiveness of pancreatic cancer cells and developed chemo resistant behaviors^3^. Therefore, in the battle with pancreatic cancer, novel treatment strategies, especially antiinvasive and antimetastatic strategies, are needed.

Juglone is a natural compound with a chemical structure of 5-hydroxy-1,4-naphthoquinone found in the leaves, roots and fruits of walnut trees and has been reported to have various pharmacological properties such as antiviral, antibacterial and antifungal. Juglone, which is an effective cytotoxic agent in various cancer cell lines, has been shown to induce mitochondrial mediated apoptosis, especially by modifying cell redox homeostasis through ROS^4^. Evidences that suggest juglone has antiinvasive and antimetastatic effects on pancreatic cancer cells are also shown in our previous study^5^.

Selenium, an essential trace element, plays an important role in many essential physiological processes by participating in the structure of selonoproteins^6^. To date, the effect of inorganic and organic Se forms on the chemotherapy of human cancers has been investigated. Sodium selenite (NaSe), one of the most studied inorganic selenium compounds, has been investigated alone or accompanied by anticancer agents. It was shown that, Se can lead to inhibit metastasis 7, reinforce cell-cell attachments (especially tight junctions of the cells) and reduction in angiogenesis^8^.

E-cadherin and integrins, located in cell membrane, are the components of the junction complexes and play critical roles in regulating cell-cell and cell-matrix adhesions of epithelial tissue and in maintaining of specific tissue architecture. Collagen is the essential component of matrix and plays a crucial role of epithelial cell attachment, polarity and maintenance of tissue function. Tumstatin, type IV collagen-derived endogenous angiogenesis inhibitor, has been shown to suppress tumor progress. Destructions of these cell junction complexes and matrix attachments cause several malignant tumor progression, affect reprogramming the tumor microenvironment and pro-angiogenesis.

In our study, it was aimed to examine the synergistic effects of Juglone and selenium on PANC-1 and BxPC-3 pancreatic cancer cells by evaluating the expression levels of CDH1, ITGB3 and COL4A3 genes and their proteins E-cadherin, β3 integrin and tumstatin, respectively, that play role in invasion and metastasis processes.

## Material and methods

### Cell culture

PANC-1 and BxPC-3, human pancreatic cancer cell lines, were provided from the ATCC (Manassass, VA, USA). The cells were cultured as indicated in our previous study^5^. Juglone and NaSe were supplied commercially (Sigma-Aldrich Chemical Company, USA).

### Viability analysis of the PANC-1 and BxPC-3 cells

MTT test was used to determine the cytotoxicity of Juglone-NaSe (abbreviated as J/S) on PANC-1 and BxPC-3 cells. Cultivation of cells in 96-well plates was carried out at a density of 5000 cells/well and the cells were treated with different juglone concentrations (0, 5, 10, 15, 20, 30, 40 and 50 µM) accompanied with NaSe (by adding 2.5 µMNaSe to each treatment group) for 24 h. Following incubation, MTT reagent was added to each well and left to incubate for four h at 37° C. After DMSO application, absorbance values were measured at 570 nm. IC50 value was calculated, four application doses (5, 10, 15 and 20 µMjuglone concentrations with adding 2.5 µMNaSe to each treatment group) were determined to investigate in both cell lines. The juglone-NaSe unapplied groups were used as the control group.

### Adhesion and invasion analysis of the PANC-1 and BxPC-3 cells

The adhesion and invasion ability of the cells was analyzed as described in our previous study^5^ by using “CytoSelectTM Cell Adhesion and Invasion Assays” (Cell Biolabs, CBA-053 and CBA-112, respectively).

### Gene expression analysis

Total RNAs isolation from PANC-1 and BxPC-3 pancreatic cancer cells was performed using TRIzol reagent and RNA quantification was done by spectrophotometer (NanoDrop, Thermo Fisher Scientific, USA). cDNA synthesis was performed using RT-PCR kit (RTPL12®, Vivantis, Malaysia). CDH1, ITGB3 and COL4A3 expression levels were analyzed by real-time PCR technique. The primers used in the analysis are shown in Table 1 9–12. β-actin was used as reference gene for normalization. PCR reaction steps were set as follows; an initial denaturation at 95°C for 5 min was followed by denaturation at 95°C for 30 s recurrently at 40 cycles, annealing at 60°C for 30 s, and elongation at 72 °C for 30 s. 2 - ΔΔCT method was applied to analyze the relative changes in the gene expressions via ‘Relative Expression Software Tool-384 (REST-384)’.

**Table 1:** Primers for qPCR analysis of gene expression

Gene	Primer sequence
*CDH1*	F: 5′-GCTGGACCGAGAGAGTTTCC-3′
	R: 5′-CGACGTTAGCCTCGTTCTCA-3′
*COL4A3*	F: 5′-AAGACCTTGGAACTCTTGGC-3′
	R: 5′-ATGTTCATTGGCATCAGAGC-3′
*ITGB3*	F: 5′-GCTTAAGGACACTGGCAAGG-3′
	R: 5′-TGGGACACTCTGGCTCTTCT-3′
*β-Actin*	F: 5′-ACTCTTCCAGCCTTCCTTC-3′
	R: 5′-ATCTCCTTCTGCATCCTGTC-3′

### Immunofluorescence analysis

The expressions of E-cadherin, β3 integrin and tumstatin were investigated by indirect immunofluorescence method at the protein level in the groups. In the immunofluorescence staining, primary antibodies for E-cadherin (Abcam; ab76055, mouse monoclonal, at 1/100 dilution), β3 integrin (Abcam; ab131056, rabbit policlonal, at 1/100 dilution) and tumstatin (Abcam; ab111742, rabbit policlonal, at 1/200 dilution) were used.

As a summary in the staining protocol; the cultured cells were fixed in absolute methanol for 10 minutes and permeabilized with 0.25% Triton X-100 solution for 15 minutes. Then, cells were kept in serum block solution for 45 minutes to block non-specific staining. After blocking, cultured cells were treated with primary antibodies for overnight at 4°C and followed by secondary antibodies (goat anti-rabbit IgG-FITC (Alexa Fluor® 488; ab150081), goat anti-mouse IgGTR (Alexa Fluor®647; ab150119) for 1 hour at room temperature. Finally, cultured cells were mounted with mounting medium containing DAPI to stain nucleus. Immunofluorescence staining results were evaluated under fluorescent microscope (Olympus; BX51) and the images (Olympus; DP72) were recorded.

The expressions of E-cadherin, β3 integrin and tumstatin in all groups were scored by dividing into 4 groups according to fluorescence staining intensity as; 0: negative fluorescence staining, 1+: weak positive fluorescence staining, 2+: moderately positive fluorescence staining, and 3+: strong positive fluorescence staining.

### Statistical analysis

Statistical analysis of the data was done via IBM SPSS 21.0 (SPSS Inc., Chicago, IL, USA) program. One-way ANOVA test was used to analyze differences between groups. The Kruskal-Wallis test was used to compare protein expression differences between groups. Mann-Whitney U test was used for paired group comparisons. In all analysis, a p value ≤ 0.05 was noted to be statistically significant.

## Results

### Juglone-selenium effects on cell viability

**Fig 1:**
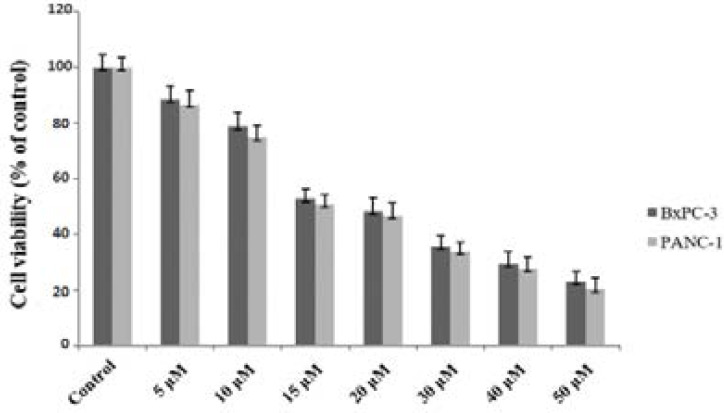
Effect of juglone-NaSe (j+NaSe) on the viability of PANC-1 and BxPC-3 cells for 24 h. Values represent the mean ±SD at three independent experiments. The IC50 doses of J/S were determined as 16.3 µM and 15.17 µM for 24 hours for PANC-1 and BxPC-3 cell lines.

Our study results showed that J/S combination inhibits cell viability in a dose-dependent manner (Fig 1). For PANC-1 and BxPC-3 cell lines, the IC50 doses of J/S were determined as 16.3 µM and 15.17 µM for 24 hours, respectively (GraphPad Prism 6 program). So, in the following experiments, J/S administration doses were decided as 5, 10, 15 and 20 µM.

### Juglone-selenium effects on adhesion and invasion properties of PANC-1 and BxPC-3 cells

**Fig. 2:**
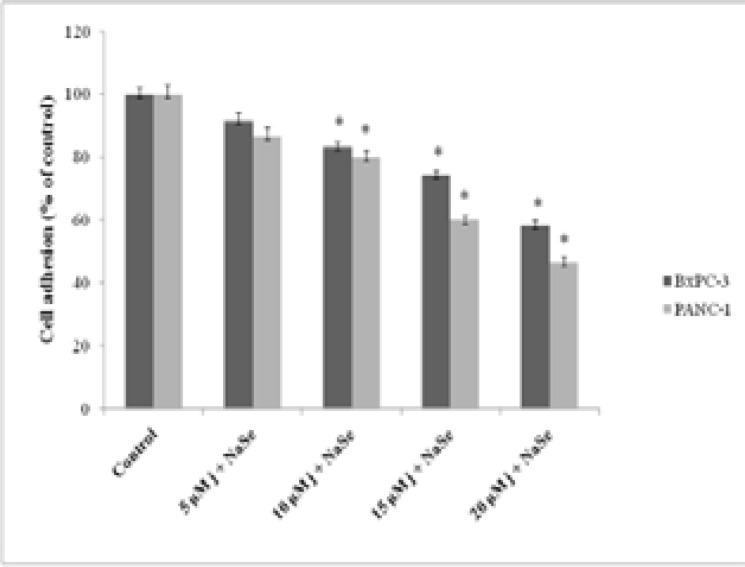
Effect of juglone-NaSe on the adhesion of PANC-1 and BxPC-3 cells for 24 h. Values represent the mean ±SD at three independent experiments. * p<0.05 compared to the control group.

Adhesion analysis results showed that J/S application decreased adhesion levels of pancreatic cancer cells in a dose-dependent manner (Fig. 2). Compared to the control, adhesion features of PANC-1 cells, were reduced by 13.4%, 20%, 40%, 53.4%, while BxPC-3 cells' adhesion capabilities were reduced by 8.4%, 16.7%, 25.8%, 41.7%. However, statistically significant effects on both cell lines were detected at 10, 15 and 20 µM doses (p < 0.05).

**Fig. 3:**
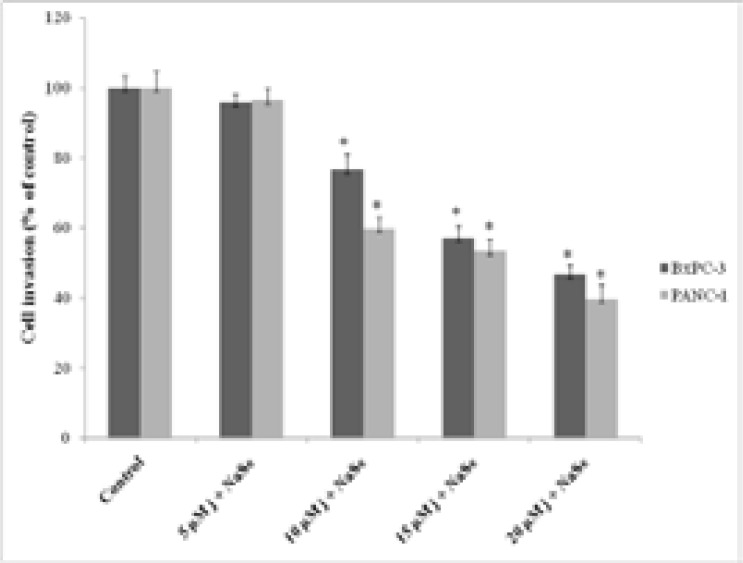
Effect of juglone-NaSe on the invasion of PANC-1 and BxPC-3 cells for 24 h. Values represent the mean ±SD at three independent experiments. * p<0.05 compared to the control group.

According to invasion analysis results, a dose dependent decrease was detected in both cell lines as shown in Fig. 3. The suppression rates in invasion ability compared to control were detected as 4.3%, 23.4%, 42.9%, 53.4% in BxPC-3 cells and 3.4%, 40.2%, 47.6%, 60.4% in PANC-1 cells. Statistically significant reducing effects on invasion in both cell lines was determined in 10, 15 and 20 juglone-NaSe applications (p < 0.05).

### Juglone-NaSe combination effects on gene expressions

**Fig. 4:**
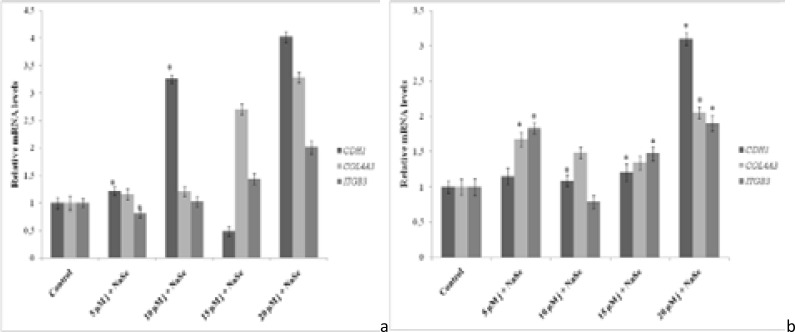
Effect of juglone-NaSe on expression of CDH1, COL4A3 and ITGB3 genes in PANC-1 cells (a) and in BxPC-3 cells (b) for 24 h determined by qPCR analysis. Values represent the mean ±SD at three independent experiments. * p<0.05 compared to the control group.

Gene expression results showed that, in PANC-1 cells, the expression of CDH1 gene rose to 1.22 and 3.26 folds in 5 and 10 µM J/S treatment while the gene expression increased as 1.09, 1.2 and 3.1 folds in 10, 15 and 20 µM J/S treatment, respectively, in BxPC-3 cells, compared to the control (p < 0.05) (Fig. 4).

While 5 and 20 µM J/S administration increased COL4A3 gene expression by 1.67 and 2.05 times in BxPC-3 cells, no significant increase in PANC-1 cells was determined for all doses (Fig. 4a).

In PANC-1 cells, ITGB3 gene expression was found significantly decreased as 1.24 fold at 5 µM J/S application (p < 0.05) (Fig. 4a). In BxPC-3 cells, 5, 15 and 20 µM J/S application caused a significant increase of 1.83, 1.47 and 1.9 fold in ITGB3 gene expression, respectively (p < 0.05), while 1.26 fold reduction in 10 µM J/S treatment was not statistically significant (p > 0.05) (Fig. 4b).

### Immunofluorescence analysis

**Fig. 5:**
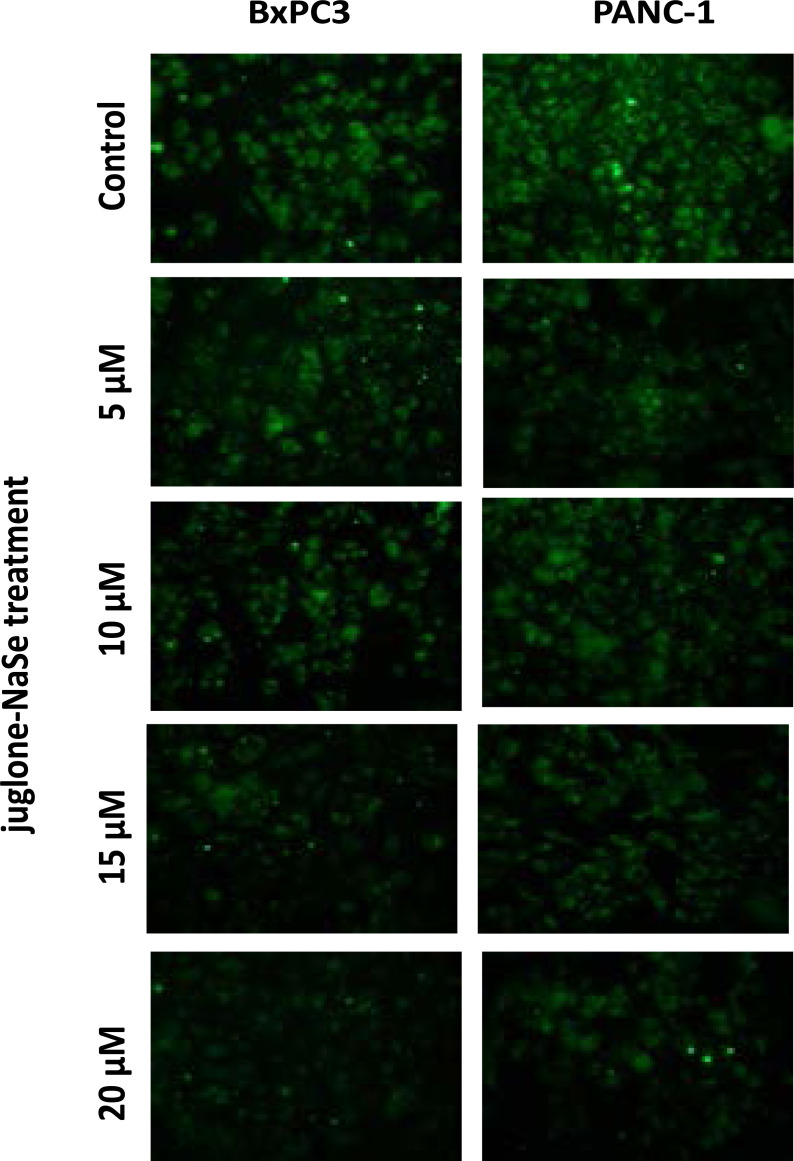
Immunofluorescence images of β3 integrin expression in PANC-1 and BxPC-3 cell lines in the groups of control and juglone-NaSe treatment at four different doses. juglone-NaSe treatment decreases fluorescence staining intensity of β3 integrin compared to the control group in both cell lines. This decrease was statistically significant at all doses in the PANC-1cell line, it was only significant at 15 and 20 µM doses in the BxPC3 line. Scales: 40 µm.

According to the results of immunofluorescence staining, E-cadherin and tumstatin expressions were not detected at the protein level in both of PANC-1 and BxPC-3 cancer cells. Moreover, J/S administration to pancreatic cancer cells at all doses did not cause an increase in the protein expressions (Fig. 5).

When β3 integrin expression was evaluated, it was observed that juglone-NaSe combination suppressed β3 integrin expression in both pancreatic cancer cell lines, depending on the application dose increase. Juglone-NaSe treatment significantly decreased the β3 integrin expression in PANC-1 cell line in all concentrations, while there was a decrease only at 15 and 20 µM concentrations in BxPC-3 cell line compared to the control group. Although 5 and 10 µM J/S treatments decreased β3 integrin expression in BxPC-3 cells, this decrease was not statistically significant. The decrease in integrin-3 expression after J/S administration indicates the inhibitory effects of J/S on invasion and metastasis ability of pancreatic cells.

## Discussion

In this study, we investigated the synergistic effects of juglone and selenium on PANC-1 and BxPC-3 pancreatic cancer cell lines by cell adhesion and invasion assays and evaluation of the mRNA and protein expressions of CDH1, ITGB3 and COL4A3 which play role in metastasis and angiogenesis processes.

E-cadherin, which is a component of the adherens junction complex, play a critical role in regulating intercellular adhesion of epithelial tissue and in maintaining of specific tissue architecture. It is known that cadherins play critical roles in tumor development and progression. Epithelial markers' down regulation (such as E-cadherin) and mesenchymal markers' upregulation (such as N-cadherin and Vimentin)^13^ are well characterized for epithelial mesenchymal transistion (EMT), which is an importnt step for the initiation of cancer invasion and tumor metastasis. The loss of E-cadherin expression about 36–60% in pancreatic ductal cancers has been reported^14^, but CDH1 deficiency and mutation are rare^15^. However, it is unclear whether the E-cadherin expression loss is a true regulator of EMT process and pancreatic cancer development^16^. The KRAS mutation is the major signature mutation in pancreatic cancer^17^. Under pathologic states with mutational Kras activation, it is suggested that E-cadherin expression loss plays a prominent role for tumor development via tumorigenic activity gain^18^. The expression of E-cadherin is complicatedly regulated by many transcription factors (Snail, Slug, Twist, WT1 and ESE3 etc) which are induced by multiple signalling pathways including Wnt, TGF-β, NF-κB and Notch 19. Thus, E-cadherin can inhibit the cancer process in many ways, including proliferation, cell migration and cancer metastasis.

In this study, we detected a dose-dependent increase in E-cadherin mRNA expression after juglone-NaSe administration in both cell lines. According to immunohistochemical analysis, there was no E-cadherin expression at the protein level in the control group and it was observed that juglone-NaSe application did not cause any enhancing effect in the E-cadherin expression. These results suggest that expression of E-cadherin is also tightly regulated post-transcriptionally. This difference between mRNA and protein expression levels of E-cadherin in metastatic cancer has also been demonstrated in previous studies^20^, ^21^. It has been noted that miR-9 can suppress the E-cadherin expression via binding to the 3-UTR of its mRNA^22^. Also, it has been shown that miR-221 inhibited expression of E cadherin by targeting the ORF of the E-cadherin mRNA transcript^21^.

In our study, we detected that 5 and 20 µM J/S administration increased COL4A3 gene expression by 1.67 and 2.05 times respectively, in BxPC-3 cells, while no significant increase was determined in PANC-1 cells. According to immunofluorescence analysis, expression of tumstatin, the protein encoded by COL4A3, was not detected in control and application groups in both PANC-1 and BxPC-3 cells. Thus, J/S had no increasing effect on tumstatin expression.

Collagen is the essential component of matrix and plays a crucial role for epithelial cell attachment, polarity and maintenance of tissue function. Collagen type IV which is a major component of basement membrane, affects the cell adhesion, migration and cell differentiation process. Collagen type IV is further organized in the basement membrane in the form of triple-helix molecules composed of combinations of six different α chain isoforms (α1 to α6)^22^. Tumstatin, type IV collagen-derived endogenous angiogenesis inhibitor, is defined as the 28 kDanoncollagenous domain (indicated as α3(IV)NC) and has been shown to suppress tumor progress in various mouse cancer models^23^. It has been observed that angiogenesis and tumor growth increase due to tumstatin deficiency encoded by COL4A3 in knock out mouse odels^24^.

In our study, we did not detect COL4A3 expression at protein level in the PANC-1 and BxPC-3 cell lines, supporting that the earlier study which reported no expression of α3(IV)NC in the pancreatic cancer cells^22^. Unlike at the protein level, we detected that two application doses (5 and 20 µM) of J/S increased COL4A3 expression at mRNA level in only BxPC-3 cells. Therefore, it is considered that COL4A3 (tumstatin) expression in BxPC-3 and PANC-1 pancreatic cell lines was not affected by J/S application.

In this study, a statistically significant decrease on the β3 integrin (ITGB3) mRNA level at only 5 µMjuglone-NaSe application was detected in PANC-1 cell line whereas the mRNA levels of ITGB3 gene was increased at 5, 15, and 20 µMjuglone-NaSe applications in BxPC-3 cells. When immunofluorescence analyses evaluated, it was shown that β3 integrin protein expression was suppressed in both cell lines depending on the administration dose increase. These findings support the dose-dependent suppression by juglone-NaSe, determined in adhesion and invasion test results.

Integrins, which are α/β heterodimeric membrane receptors, mediate cell attachment to the extracellular matrix. In humans, there are 18 alpha and 8 beta subunits that combine in pairs to form at least 24 different α/β integrin heterodimers^25^. β3 integrin is one of the most studied members of the integrin family, which plays several crucial roles such as in malignant tumor progression, in reprogramming the tumor microenvironment, in stemness regulation and in pro-angiogenesis^26^. So, integrin-suppressing molecules are seen as attractive agents for targeted therapy approaches in cancer. As an αvβ3 integrin-specific inhibitor, cilengitide was the first candidate antiangiogenic drug and is progressed in phase II clinical trials which showed encouraging outcomes in pancreatic cells, glioma, and some metastatic solid tumors^27^, ^28^. However, further studies are needed because of cilengitide did not show a significant improvement in phase III studies^29^, ^30^. Understanding the multiple roles of β3 integrin in the tumor microenvironment is essential to assist directly in the design of specific targeting strategies to maximize clinical effects^26^.

In pancreatic cancer, late diagnosis and chemotherapy failure are the two major problems for treatment success and overall survival^31^. The limited antitumor effects of chemotherapy and radiotherapy treatments alone, and the lack of approved targeted therapy to date, indicate the urgent need for new approaches to increase the effectiveness of chemotherapy^32^. In recent years, researches have been focused on targeted combination therapies for advanced/metastatic pancreatic cancer^33^. In most of those researches, several natural products/derivatives were investigated for pancreatic cancer treatment^34^, ^35^ and mechanisms such as cycle regulators, DNA repair mechanisms, epigenetic change players, signaling pathways and targets, epithelial-mesenchymal transition and metastasis markers, apoptosis and autophagy were targeted. In our opinion, antiangiogenic, antiinvasive and antimetastatic key molecules are the critical targets in the studies to investigate new treatment approaches for pancreatic cancer characterized by early metastasis.

In our previous study^5^, for the first time we demonstrated the cytotoxic effect and also antiinvasive and antimetastatic features of the juglone in PANC-1 and BxPC-3 pancreatic cancer cells by adhesion/invasion test and evaluating MMP-2, MMP-9 and Phactr-1 gene expressions which are important markers for metastatic process. In this study, we aimed to investigate the effects of juglone and selenium combination on cytotoxicity, adhesion and invasion process in PANC-1 and BxPC-3 cells. Our current study showed that juglone and selenium combination has a stronger cytotoxic effect than juglone compared to the results of our previous study, suggesting that probably selenium strengthens the cytotoxic effect of juglone. Also, our study has provided evidences that the combination of juglone-selenium has a dose dependent suppressive effect on adhesion and invasion capabilities of pancreatic cancer cells. We suggest that this combination shows its effects via regulating β3 integrin expression at posttranscriptional level. Consequently, our results showed that juglone-selenium combination has a cytotoxic and antimetastatic effect on human pancreatic cancer cells. However, more indepth studies are needed to demonstrate the effects of juglone-selenium more clearly, which has the potential to be a promising agent especially to inhibit invasion and metastass in pancreatic cancer treatment.
